# Biomarkers and their involvement in the early diagnosis of right ventricular dysfunction in type 2 Diabetes Mellitus

**Published:** 2012-03-05

**Authors:** O Vittos, B Toana, A Vittos

**Affiliations:** “Carol Davila” University of Medicine and Pharmacy, Bucharest, Romania; MedCenter, Bucharest, Romania; “Dr. Carol Davila” Central Clinical Military Emergency Hospital, Bucharest, Romania

**Keywords:** lipoprotein associated phospholipase A2 (Lp-PLA2), Diabetes Mellitus type 2, Strain and strain rate Right Ventricle

## Abstract

**Objective:** During the last years, left ventricular dysfunction in diabetes was intensely studied and it is recognized as a complication of diabetes, while data regarding the right ventricular dysfunction is still incomplete. The aim of this study was to find a correlation between inflammatory biomarkers, adiponectin and right ventricular strain and strain rate properties in patients with diabetes mellitus type 2.

**Methods:**We studied 29 patients with type 2 diabetes mellitus (DM), with coexisting cardiovascular complications, coronary artery disease and high blood pressure (group 1- DM+CV, aged 61.2 ±4.2 years); and 22 patients with type 2 diabetes and controlled high blood pressure only, with no other coexisting cardiovascular complications (group 2- DM, aged 60.86 ±4.4 years).

We evaluated the right ventricular (RV) function through Vector Velocity Imaging (VVI)and determined the inflammatory profile through assessment of the following biomarkers: high sensitivity C- reactive protein (hsCRP), tumor necrosis factor-alpha (TNF-alfa), lipoprotein associated phospholipase A2 (Lp-PLA2) and adiponectin level for each patient.

**Results:**VVI revealed significantly lower values of systolic strain and strain rates (SR) in the basal segment of the RV free wall in group I patients (DM+CV) as compared to group II patients (DM) which indicates higher impairment of RV systolic function in patients with diabetes and other cardiovascular complications. In both groups strain and strain rate values were correlated with Lp-PLA2 activity levels. Conclusions:In Type 2 diabetes mellitus we identified a low-grade inflammatory status correlated with right ventricular systolic dysfunction.

## Introduction

Type 2 diabetes mellitus (DM) is characterized by a low-grade inflammatory status and endothelial dysfunction, which substantially potentiates the risk of developing cardiovascular diseases. The impact of diabetes leads to myocardial dysfunction, which is initially sub-clinical and might lead finally to diabetic cardiomyopathy and heart failure (HF).

In the latest years, a lot of evidence has been collected regarding left ventricular dysfunction as a common complication of diabetes mellitus [**1**], while data regarding right ventricular (RV) performance and the impact of different inflammatory factors on its performance, is still incomplete. 

It becomes imperative to identify some biomarkers footprints, which could reveal the asymptomatic patients at high risk of evolution to left and right ventricular myocardial dysfunction, making effective prevention possible. 

Right ventricular function, has been recognized as a significant indicator of clinical outcome and prognostic value in heart failure, myocardial infarction, pulmonary embolism and more recent in diabetes, too. [**2-5**].

It is already known that lipoprotein-associated phospholipase A2 (Lp-PLA2), is a risk marker for endothelial dysfunction in patients with type 2 diabetes and recent data suggest that Lp-PLA2 might be a biomarker constantly correlated with HF, regardless of aetiology [**6-7**]. Elevated plasma values of Lp-PLA2 in heart failure with preserved ejection fraction (HFpEF) are consistent with the exacerbated inflammatory status [**8**].

Recent research proved that adiponectin (Adpk) is correlated with the presence of atherogenic dyslipidemia and with N-terminal prohormone of brain natriuretic peptide (NT-proBNP) concentration but not with markers of systemic inflammation such as IL-6 or hsCRP in patients with manifested coronary heart diseases [**9**].

Data collected from current research indicate diabetic disease-specific alterations in the biochemical parameters, Adpk level is inversely correlated with hsCRP in end-stage renal disease patients [**10**].

In this study, we have evaluated Lp-PLA2 activity, Adpk, TNF-alfa and hsCRP, in relation with the right ventricular parameters and tried to determine the initial, asymptomatic effects of DM and high blood pressure on the right ventricular systolic function. 

In addition, Lp-PLA2 activity and levels of Adpk, TNF-alfa and hsCRP, correlated to, or preceding echocardiographic findings, may improve the early diagnosis of cardiovascular complications in diabetic patients.

## Methods

### Study population

The study included 51 patients with diabetes mellitus type 2 (DM type 2), screened between Jan 2010-March 2011. Patients were divided into two groups: group 1, consisting of 29 patients with DM type 2, high blood pressure ( HBP) and known coronary artery disease (CAD) and group 2, consisting of 22 patients with DM type 2 and HBP, without CAD. For group 2 patients, CAD was excluded by negative results on treadmill test. 

Other exclusion criteria were: pulmonary hypertension, HF class III-IV NYHA, Left Ventricular Ejection Fraction (LVEF) < 45%, renal failure, hepatic failure, recent stroke, Transient Ischemic Attack (TIA) or acute myocardial infarction in last 6 months, absence of sinus rhythm, neoplasm, patients taking anti-inflammatory medication and poor quality of echocardiographic parameters. The characteristics of the studied population are presented in **Table 1**. 

Patients underwent clinical examination, routine laboratory tests, resting or treadmill test ECG, echocardiography (Velocity Vector Imaging- VVI). The treadmill test was performed according to the Bruce protocol using a positive response defined as the occurrence of at least 1-mm ST segment depression in comparison with the basic line tracing. Patients with BMI over 30 were considered obese. HBP was defined as documented systolic blood pressure ≥140 mmHg and /or diastolic blood pressure μ90 mmHg, or by having anti-hypertensive treatment ongoing. DM type 2 was controlled by oral anti-diabetic medication and specific diet. Concomitant medications consist of: ACE inhibitors, statins, beta-blockers, calcium channel blockers, aspirin, clopidogrel, nitrates, as per their specific diagnosis. Echocardiography was performed on Acuson Sequioa System 256 (Siemens). 

The VVI concept is based on the fact that during a cardiac cycle, tissue moves from one point to the next, and by multiple speckle tracking algorithms we are able through VVI to identify the tissue movements at every point, allowing analysis of strain and strain rate (SR), in longitudinal, radial or circumferential directions. In our study, we followed only the longitudinal parameters and we draw one trace on the RV endocardial border at end-systole, followed by the automatic endomyocardial border tracing throughout all cardiac cycle. For strain and strain rate measuring, we averaged three cardiac cycles with a frame rate set at 31 frames per second. Regional parameters were calculated for RV for the free wall, basal and mid points, from an apical four-chamber image. 

All plasma samples were frozen and stored at -70°C- until analysis. Lp-PLA2 activity was measured using the spectrophotometric method described by Kosaka et al. using the Azwell Auto PLA2-LDL kit [**11**]. Enzymatic activity is expressed through IU/L. Adpk, hsCRP and TNF-alfa, were determined through ELISA.

**Table 1:**
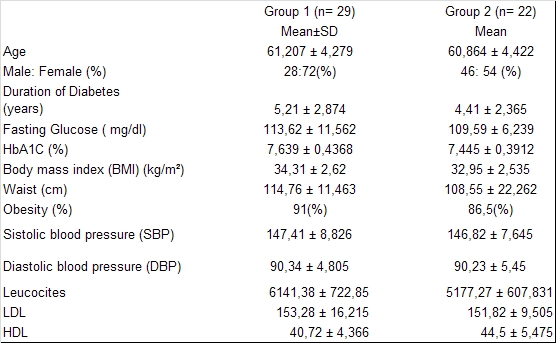
Characteristics of studied population, in investigated groups

### Statistical Analysis

The statistical analysis was performed with the SPSS statistical package. The values presented are mean values ± SD. The following statistical tests were used: Student’s two-tailed t-test, ANOVA, Pearson and Spearman rank correlation tests, linear regression. P value of <0.05 was considered statistically significant. 

## Results

In group 1, patients with type 2 diabetes and cardiovascular disease, the Lp-PLA2 activity was significantly higher, with mean value (SD) 419.46 UI (76.21), compared to values detected for patients with diabetes and without CVD (group 2), where Lp-PLA2 activity mean value (SD) was 307.22 UI (19.01). As noticed also in other studies, values of CRP and TNF-alfa, were higher in group 1 (DM+CVD), than in group 2(DM only). (**Table 2**) For Adpk there was none correlation identified with any investigated parameters in group 1 patients. In group 2, we identified a negative correlation with SbasalVR (p<0.05) as well as a negative correlation with Lp-PLA2 (p<0.01). For both investigated groups, CRP values were not correlated with any RV echocardiographic parameters, but for CRP it was noticed a positive correlation with TNF-alfa for both groups (p< 0.05). And in addition, for group 2, we noticed a positive correlation between hsCRP and Lp-PLA2 (p<0.01).

**Table 2:**
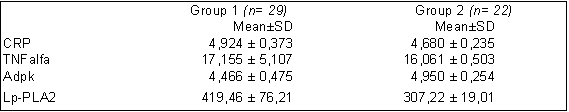
Mean value and standard deviation for CRP, TNF-alfa, Adpk, Lp-PLA2, in investigated groups

Lp-PLA2 activity was significant statistically negative correlated with LVEF in both groups (p<0.01) and positive correlated with RV investigated parameters (p<0.01).

**Figure:**
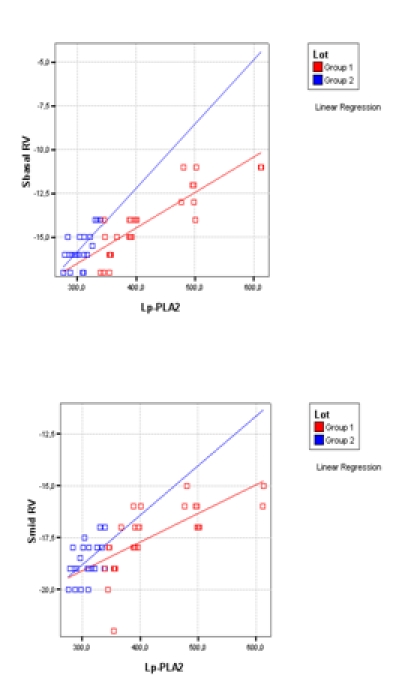


We found significantly lower values of systolic strain and strain rates in the basal segment of the RV free wall in group 1 patients (DM+CV) as compared with group 2 patients (DM) indicating higher impairment of RV systolic function within patients with diabetes and other cardiovascular complications. (**Table 3**)

**Table 3:**
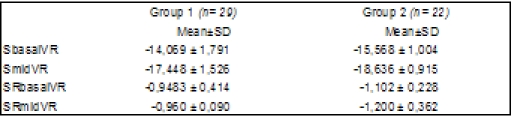
Mean value and standard deviation for S/SR for basal and mid RV free wall, in investigated groups

We identified significantly differences between groups for hsCRP, Adpk, SbasalRV, SmidRV, SRmidRV and Lp-PLA2 (p <0.01).

## Discussions

According to the World Health Organization (WHO), the cardiovascular diseases are the number one cause of deaths worldwide, responsible for over 30% of all global deaths annually. Diabetes is known as a risk factor for CVD, the risk being increased by 2 to 4 times compared with adults without diabetes, and this determines the development of heart failure, sometimes even in the absence of its frequently coexisting comorbidities HBP or CAD. 

In the last ten years, there has been a lot of evidence accumulated regarding left ventricular dysfunction (systolic and diastolic) in diabetic patients; however, less research has been done for identifying the right ventricular dysfunction. A recent study investigated left ventricular strain and strain rates in diabetic patients. This study concluded that left ventricular longitudinal systolic and diastolic functions were impaired, while the circumferential and radial functions are preserved in patients with uncomplicated type 2 diabetes mellitus [**1**]. Also, a study done in type 1 diabetes mellitus patients proved that before any development of systolic dysfunction, the diastolic performance is altered, in both ventricles, suggesting that these alterations in myocardial function could be attributed to ventricular interdependence as well as to the global effect of diabetes on cardiac function [**12**]. 

Until now, none study has been published regarding relation between inflammatory biomarkers and right ventricular strain and strain rate in diabetic patients. We have included in our evaluation the Lp-LPA2 activity enzyme which is considered a link between lipid metabolism and low-grade inflammation and characteristic marker for cardiovascular or metabolic diseases, being considered also a valuable predictor of CVD. The West of Scotland Coronary Prevention Study (WOSCOPS) demonstrated that high levels of Lp-PLA2 were associated with a two-fold increased risk of CHD. This relationship was valid also after taking into account the traditional CV risk factors and other inflammatory markers (including hsCRP) [**13**]. It is worth mentioning that recent research has identified that Lp-PLA2 may be in fact superior to other inflammatory markers, such as hs-CRP, due to its specificity and minimal bio-variation [**14**]. In addition, Lp-PLA2 is now recognized as a marker of oxidative stress and vascular inflammation which has a relative unique characteristic being independent from BMI and insulin resistance [**15**]. 

In our study, the higher values obtained in group 1 patients (DM+CVD) for Lp-PLA2 (p<0.01) are in correlation with the inflammatory state characteristic of those patients. Furthermore, we found a significant but modest inverse correlation to HDL cholesterol, a finding consistent with many studies [**16,17**], but not all [**18**]. Interestingly, hsCRP did not correlate with any RV echocardiographic parameters, while Lp-PLA2 proved strong positive correlation with all investigated parameters. For group 1 patients ( DM+CVD), Adpk did not correlate with any investigated parameters of right ventricular dysfunction, a finding consistent with other studies [**19**]; while for group 2, we detected a negative correlation SbasalVR (p<0.05) as well as a negative correlation with Lp-PLA2 (p<0.01). This finding might suggest that for uncomplicated DM, Adpk might be a potential marker of underlying mechanisms linking the diabetic state to cardiac abnormalities [**20**].

We noticed a higher impairment of right ventricular longitudinal systolic function in group 1, compared with group 2, being statistical significant for SbasalRV, SmidVR and SRmidVR (p<0.01). 

Similar results regarding RV echocardiographic parameters in diabetic patients were recently detected through 3D echo, stating the right ventricular systolic and diastolic dysfunction in diabetic patients [**21**]. 

By assessing the inflammatory profile of diabetic patients, it has been revealed that, even those asymptomatic for CVD, have a continuous inflammatory state, together with a decrease in RV systolic function. 

### Limitations

Due to angle dependent strain measurements, there should be caution in interpretations of strains when tissue direction deviates more than 30° from the beam direction. This could be a significant limitation of this technique, and reposition of transducer might help to correct this issue. Another limitation of this technique is the normal beat-to-beat variation in stroke volume that could explain why strain profiles do not always return to exactly same point of baseline at end diastole. That’s why for this study we averaged three cardiac cycles. 

Also, this is a cross-sectional study, with data collected at a given time-point, and due to this, it could not provide prognostic value of inflammatory markers. It is necessary to extend the study with a follow-up period.
